# On the Forms, Causes, Sanability, and Treatment of Pulmonary Consumption

**Published:** 1832-10-01

**Authors:** 


### VIII.

### A PRACTICAL TREATISE ON THE FORMS, CAUSES, SANABILITY, AND TREATMENT OF PULMONARY CONSUMPTION. By *Edward Blackmore,* M.D. Physician to the Plymouth Public Dispensary. pp. 242. London, Longman and Co. 1832.

IN devoting a page or two of our Journal to the notice of the present work, by Dr. Blackmore, we are influenced by two considerations; one of which is more immediately applicable to the author—the other has reference to the public. An advertisement is appended to the volume, announcing that a second may be soon anticipated by his readers, its materials being ready for the press. Prompted by the most friendly feelings, we advise the author to rest a little from his labours, till he ascertains the amount of fame and of profit that may accrue from his present undertaking. It is not because a volume contains two hundred and forty-two pages, sufficiently well-printed and neatly got up, that, therefore, there must be something valuable—something new, or at least newly-illustrated and arranged, in its contents. —One might naturally expect this to be the case; but we are soon to be undeceived. So easy it is to become an author in the present day, that any boy, before he leaves college, may, by industriously copying out a few cases of any particular disease from the clinical records of a hospital, and affixing to the details some threadbare memoranda or flimsy remarks, have his name blazoned in all the newspapers and advertising-sheets of the Mæcenases of the age, and associated, at least for a month or two, with the names of Abercrombie, Bright, and other celebrated names.

> " 'Tis pleasing, sure, to see one's name in print,
> A book's a book, altho' there's nothing in't."

The judgment of the poet may be correct in the opinions of booksellers, of the authors themselves, and the authors' friends and patients; but, as reviewers, we may find that we cannot agree with them. Our duty to the medical public is twofold—to deliver a candid and impartial opinion of the general merits of the books sent for our inspection, in order that our professional brethren may be guided in the selection of such works as are useful, or essential to have in their libraries; and, secondly, to extract the most important and useful information from their pages; to concentrate the pith and marrow of their contents.

A very copious preface meets us at the threshold, in which the author descants upon the " inductive" and " eclectic" methods of writing ; but we confess ourselves incompetent to understand the interpretation which he gives of them. Then are adduced extracts from d'Alembert, Galen, Haller, Bacon, Horace, Boethius, Cullen, Pinel, Friend, and a host of others, to prove or illustrate we hardly know what.

The second section of the preface treats of the various definitions and divisions of consumptive diseases; the nosological, pathological, and noso-pathological modes of classification, which, we are assured, "are harmonious, and complemental of one the other." The following two sentences are extracted from one page, and we must leave it to the ingenuity of our readers to make them, if possible, harmonious and complemental.

"The symptoms *alone*, on the other hand, cannot be made the ground-work of pathological distinction, for under similar symptoms we find several organic lesions, various in appearance, but similar in kind." "The forms of pulmonary disorganization which are pathologically different, will be found to exhibit a cognisable diversity in the combination or succession of the symptoms; although that diversity may escape the perspicacity of careless observers." xvi.

The third section is occupied with numerous extracts from several authors, strung together with somewhat random *profusion*, if not sometimes *confusion*. Baglivi, Alison, Bishop Berkeley, the Edinburgh Review, Dr. Baron, and Dr. Whately (from whose work on rhetoric we are favoured with a long quotation on "what constitutes experience"), are the names which figure in Dr. B.'s pages.

The preface, which occupies no fewer than 24 pages, being thus discussed, we proceed with our analysis. The very first sentence of the first chapter has these words:

"The Pathology of the Lungs comprehends three genera of disorders; the Inflammatory, the Consumptive, and the Nervous. The Consumptive Affections include all the cachectic* disorders which are characterized by cough, purulent sputa, hectic fever, and emaciation." 1.

Now, if consumptive diseases are quite distinct from inflammatory ones, how will the definition quoted from Mason Good be upheld? for we are told that consumption is a cachectic disorder, and that a cachectic disorder is "a depravity which originates from, or manifests itself in, the sanguineous function, as it regards the vessels or the fluid." Are not inflammations depravities, that is, abnormal changes, or aberrations from a healthy state, in the sanguiferous system? But, waiving this objection which more pertains to critical accuracy than to practical importance, is it possible that Dr. Blackmore has never seen a case of unequivocal phthisis, accompanied with symptoms of obstinate inflammation during life, and exhibiting undisputed proofs of the result of inflammation after death? Does he not know that an attack of pneumonia or of pleuritis, in a person hereditarily disposed to consumption, will often, very often, lead on the melancholy train of hectic fever, cough, and purulent expectoration? These three symptoms our author considers as pathognomonic of the disease; and so widely comprehensive is

* "The term Cachexy is used to signify 'a depravity which originates from, or manifests itself in, the Sanguineous function, as it regard the vessels or the fluid.' Dr. Mason Good, physiol. nosolog. p. 223. All organic diseases are properly herein comprised."

his definition of consumption, that he admits even " fatal cases of organic disease in the abdominal viscera, with cough and purulent sputa ;" and yet, in his preliminary classification of thoracic diseases, he adopts the most palpably-erroneous arrangement on which an author could well stumble ; namely, into such as are inflammatory, consumptive, and nervous. In which of these classes or orders is the learned author to find a plaoe for hæmoptysis, œdema, and emphysema of the lungs, pulmonary apoplexy, and so forth? We allude to this fatal error, occurring, too, at the very commencement of the book, as exhibiting the unpardonable negligence with which it has been got up. Proceeding, step by step, we find ourselves lost in a forest of names, to each of which is tacked a disjointed sentence; and when we have at length emerged, we discover that there are two orders of consumption, the " spurious" and the " genuine."

" That is genuine Phthisis, in the language of Celsus, when decline originates in a disease of the Lungs ;—and the epithets Tabes, Atrophia, Marasmus, are used by recent nosologists to designate consumption from disease in other organs. When the lung is affected secondarily, and its disorder in itself minor and innocent, disease in another part being the primary affection, and the essential cause of death, such a malady may be properly designated Spurious or Secondary phthisis : and a diverse order of succession in the symptoms will distinguish this case from the primary idiopathic affection of the Bronchial membrane which is termed Mucous or Catarrhal Consumption." 6.

And again—

" If it is correctly assumed that phthisis is essentially a *fatal* pulmonary affection, a case of hectic from suppuration in the lungs is not truly phthisical ; for such a condition is *sanable*; this may be styled spurious consumption, in distinction from those cases which are necessarily mortal. To apply the term, however, to an insanable case of pulmonary disorder with phthisical symptoms, because the lungs are *not* visibly disorganized, is to introduce confusion into Nosology and Pathology." 6.

And again—

" The general arrangement adopted in this Treatise, will exhibit TWO distinct ORDERS ;—First, the cases with the usual symptoms of consumption, apparently hopeless, but ending in *recovery*. (See chap. III.) And Secondly, the cases progressively passing on to a fatal termination. In some of the cases in this latter division the disorganizing malady is seated in other organs than the lungs; the cough and puriform expectoration being secondary. In these, the supervenient pulmonary affection (which in most instances is confined to the mucous membrane of the Bronchia,) is not the proximate cause of the hectic and emaciation ; nor is it essentially fatal: the primary disorder, however, has often been so completely disguised by the supervenient affection as only to have been detected on dissection.—(See Dr. Hastings on *Bronchitis*, p. 117.) An organic disease in the abdominal viscera may subsist in a latent or tranquil state for years; and then on the rise of a slight pulmonary disorder the patient will be rapidly cut off: so that this secondary affection, which by itself would be innocuous, will in this complication be of important consequence." 8.

Bewildered and confused as we have been in the maze of errors and inconsistencies, we scarcely, however, expected to find that one of the forms of consumption is designated chronic, remittent, and *inflammatory*, after the boundary line had been drawn in the memorable first sentence of his book! To render the inconsistency still more inexcusable, read the following sentences, which the author indites preliminary to the detail of cases exemplifying the first form.

"Two Orders of cases will be given ;—First, those which were seen in a state of pulmonic inflammation, but ascertained by their result to be of a truly consumptive nature, although life was destroyed before the completion of the phthisical disorganizing process ; and Second, cases seen in the state of confirmed phthisis. In some the affection was chiefly seated in the bronchial membrane, or in the interior of the lung,—consisting in a suppurative action, with or without Tubercles ; in others, the symptoms indicated an affection of the exterior or Serous membrane of the lung and of the chest. The former may be styled Empneumonic, the latter Peri-pneumonic cases. Some cases again are simple, the Lungs alone being diseased ; in others, there is an important complication of other maladies." 11.

"The phthisical inflammation may be regarded, *first,* as to its *seat* in the substance of the lung, or in its serous membrane ; *secondly,* as to its distinct nature in the several forms of consumption. 1.—'There is a variety of peripneumony where there is no pain, which is longer and severe,—the lung is very dark.'* There is a similar fallacy in the symptoms of this variety to that which Professor Burns has remarked of Bronchitis in infants,—'a *pale* face, and oppressed look, which delude into a trifling mode of cure.' It is important not to be deceived by a low oppressed pulse.† There is a practical observation of Huxham worth remembering, that in pneumonic inflammation, pain at the bottom of the chest, with borborygmi, and a swelled belly, is relieved by clysters,'—the intestines being the seat of disorder." 215.

We deem it quite unnecessary to select any number of cases from the present work ; they have been fished from all possible sources, and are reported with such prolixity, as to render them tediously uninteresting and little instructive ; every minute particular is wearisomely enumerated, and we sometimes in vain search, in the report of the dissections, for the pathology of the disease in question. Strange that our author should be guilty of an error, which he reprobates himself in the words of Haller, quoted in the preface :

"Sincere et simpliciter enarrare quæ vidi, et breviter ; vereor enim ne parum ii scriptores legantur qui nimia abundantia *exiguorum accidentium* historias suas producunt, et lectuis tædium movent." viii.

But to satisfy our readers that the above strictures are warranted by impartial justice, we shall allow them to judge for themselves of the followiug cases, which are transcribed verbatim.

"Case XIII.—A. T. a 22, (Jan. 1831,) of high sensibility and genius, in June, 1829, after a severe cold, was affected with cough, and pain at the left chest, which was neglected for three months. In the winter she suffered much from fatigue and mental anxiety. In the spring of 1830 she spat blood, and the pain of the side became acute. Leeches and blisters were then used, general bloodletting being neglected : the cough persisted during the summer, with expectoration of one ounce of matter in a week, like *soft cheese* mixed with common mucus, which on drying became friable ;—the matter of Tubercles ? A physician then thought her to be in consumption, and prescribed the lichen island. and change of climate. She has never worn flannel ; and has persisted in the use of wine and animal food. There is now a deep sonorous cough, occasional

---

* Dr. Hooper. † Dr. D. Monro.

hoarseness of the voice, evening fever, and some emaciation ; the menstrua irregular. 6th to 11th—An attack of Measles, during which the cough and sputa were suspended, and diarrhœa came on. Kino, creta, and flannel were then prescribed. After the measles severe pain at the side returned, with a harsh cough, and difficult expectoration. (Nitrat. potass. liq. ammon. acet. tr. digit. pil. hyos. p. Jacobi, ipecac. hirudines.) 14th—Respiration very quick, costiveness, a rapid but softer pulse, (110—120) a loaded dry tongue, intense thirst. (Calomel. scammon. tr. scillæ. ung. hydr. antim. tart.)—17th—Cough less harsh, but in severe paroxysms at night with more excretion of viscid opake bloody mucus, without a vestige of pus, or tuberculous matter; four stools a day, which are more natural; hectic at 2, p.m. pulse 120, tongue clean and moist. (Hyosciam. scilla, cum aceto.) 18th—Cough relieved; some blood from the nostril. Percussion painful below the clavicle, and betwixt the shoulders, where also by the stethoscope the respiration is inaudible. The voice is so raucous and low that pectoriloquy cannot be ascertained. (Acid. nitric-hydrodyanic. narcotic, h. s.) 19th—The narcotic has been of admirable effect; hectic at noon, during which there is oppression at the lungs, with hard cough and laborious respiration, and appetency for solid food. Interm. acid. nitric.-hydrocyan.) 20th—Good stools, pulse 110; defect of voice remains, but no irritation at the larynx. (E. C. hyos. cum digital. vesp.) 21st—Fever and cough relieved, sputa less viscid, tea vomited, but no nausea; pulse 100. (Acid. nitric. victus ex carne decocto.) 24th—Cough severer, sputa more copious, ragged and viscid, tongue loaded, the legs ache, vertigo on exercise, much emaciation, no hectic, soreness at the larynx, fetid stools. (E. c. alia ut 6to. supra.) 27th—Since the severe weather the symptoms have become worse, the arm is numb as if the ulnar nerve were struck; some roast chicken eat. (Acet. scillit. hyosc. &c.) 28th—The sputa sink in water, are fetid and caseous, seeming to consist of pus or soft tuberculous matter; hectic high, with tightness at the chest, which some leeches relieved, pulse 100.—The adhesive inflammation in the lungs seems to have prevented the opening of the tubercles into the bronchia before now; the inflammatory stage, which began in the measles, has ended in a month. 29th—The caseous sputa enveloped by viscid mucus. (Infus. calumb. aurant. acid. nitric. prussic t. d. hyosciam. scilla. o. n. pil. cath.) 31st—More cough, and fawn-coloured sputa; pulse 120, soreness beneath the sternum. (Omitr. victus ex-carne, et tonica. St. scil. et digital. hiruds.) Feb. 1 and 2, relief. (E. c.) 3rd —Pulse 130; less pus and blood in the sputa. (Infus. humuli cm. digital. t. d. liq. opii sedat. hyosciam. ipecac. o. n. vegetable diet.) 10th to 14th—Stupor and delirium from the narcotics; the sputa now consist of pure pus; aphthæ and diarrhœa, tremors from debility, pulse 110 to 130. (Infus. calumb. cm. aceto. liq. calcis, catechu, acet. morphiæ.) Diet of yolk of egg and beef tea.

By these remedies the diarrhœa was stopped, the thrush disappeared, and the system was composed, but copious purulent cream-like sputa, with colliquative sweats, continued.

19th—Mild delirium from exhaustion, after transporting religious emotion. On using some *cold* lemon-tea, she complained of a sense of suffocation as if dying; this was relieved by external heat; pulse very small and rapid. (Claret ordered.) 22nd—Aphthæ have recurred; much cough, less easy expectoration; the sputa are large, and seem to come from large cavities in the lungs; little fever. The subsidence of fever, when fits of syncope and suffocation supervene, marks the approach of death. 25th—Porter and animal jelly used, little medicine but magnesia and rhubarb; more cough, hectic and debility; dyspnœa in paroxysms. (E. canth.) 26th—Relief of suffocation, but the odour of death observed all the day. At 6, p.m. mortal exhaustion ensued on her making a slight effort; at 7, consciousness was entire, her mind full of christian peace and hope; then followed a dreadful agony of suffocation, which was relieved by admitting the fresh air; at eight o'clock the pulse was gone and the limbs cold; then mild delirium and risus sardonicus, which were succeeded by coma, the respiration continuing, but performed chiefly by the auxiliary respiratory muscles, then it became suspended at intervals; shortly after a rattle in the throat the last breath was expired, her countenance remaining beautifully tranquil;—a faint emblem of the bliss of her saintly spirit in the presence of her Saviour and her God!

The measles certainly excited into fatal action the disease in the lung, which had probably long ago formed tubercles and induration. The softening and evacuation of tubercles is here seen to be a distinct condition of the lung from inflammation; although this is often an adjunct which essentially promotes that state. The symptoms in January were obviously distinct from the simple debility hectic and purulent expectoration which existed during February: the change observed in the sputa from a viscid to a cream-like character marked the entire subsidence of inflammation. The utility of sedatives, narcotics, and counter-irritants was very manifest. The unusual definiteness and alternation in the symptoms and the phenomena of dissolution render this case very interesting.

Case XIV.—Horral, m. a. 30 (*February* 20, 1829), of a florid strumous aspect, has been ill for six weeks, from cold, with cough, sputa and much dyspnœa. He has had diarrhœa lately; at present tremors, a white tongue, and rapid pulse. Several of his relatives have died of phthisis. (Colchic. scilla, digitalis, creta cum opio.) 24th—Much better. (Acid. nitricum, rheum, E. C.) *March* 4th—No cough, but some pain at the side, and breathlessness on exertion, a quick pulse and a white expanded tongue. 11th—Much breathlessness; aspect delicate. (Sulphas ferri, digital.) 16th—Cough, perspiration, red urine, a rapid pulse; in other respects better. (Sulphas potass. cum rheo, scilla, et alia.) Pain at the stomach like heartburn, ascribed to the medicine; little cough, mind dejected. (Magnes. E. Canth.) 30th—Cough, with breathlessness and fainting, but the stomach is better. (Scilla, æther, digital.) *April* 6th—Complains of heat at the stomach and sleeplessness. (Magnes. digit.) 8th—Vomiting and ischury. 15th—Phthisis advancing; the legs swell, and he is sick and giddy; all the medicine, even soda-water, is vomited; gastritis erythematica developed, hectical aspect. (Nitric. acid. digitalis, hirud. magnes. in aq. cinam.)—The mildness of the phthisical symptoms, and their alternation with the dyspeptic, deserves remark. 20th—Much anasarca. (Acetum scillit.) 26th—Death.—The disease ran its course in four months.

A well marked instance of the acute scrofulous consumption; it forms an interesting subject of comparison with Langdon's and Urquhart's; the tonics were here also injurious. *See* Louis, in chap. II. for the morbid appearances in the stomach in similar cases."

"Complicated with Disease in the Brain.

Case LXI.—Rendel, m. a. 60, of a tense spare habit, and temperate, (Aug. 1829,) has long had pain at the head, vertigo, and a gradual loss of vision, more entire in the *right* eye, which is small and retracted, while the *left* is large and protruded. In September he had much aching through the forehead; complete blindness on the right eye, but the pupil was moveable, the sight of the left imperfect. Various means were used at the Eye Infirmary without effect. At the end of Oct. he had a severe attack of pain in the right side, for which he was once bled; he then went into the Poor-house Hospital, where cough and dyspnœa continued, and the disorder in the head, with low nightly delirium, increased. Dec. 8th—Little cura adopted;—death this morning.

*Inspection*—10 hrs. p. m.—Much blood in the integuments of the head, the inner table of the frontal bone at the sinuses and crista galli was widely separated from the exterior, and inclined inwards on the brain; the left orbitar plate destroyed; the back part of the orbit filled by a tumor the size of a large walnut, of a cystic honey-comb structure, of a bony hardness, its cells small, filled with fluid like white of egg and honey; it had nearly corroded the left temple, and extended into the posterior nares; through it passed the third pair of nerves, enlarged and softened; the optic nerve which also passed through its lower part was small; as was the eye-ball, but not evidently diseased. The tumor adhered by a glutinous substance to the temporal bone and to the eye-ball; appearing to have corroded all the parts it touched; it extended to the right orbit, the plate of which was partially carious; all the processes of the ethmoid bone and the vomer were carious, or softened, so as to be easily cut out. The brain and cerebellum small, soft, bloody points in it; 1 oz. of serum in the ventricles; a small hydatid in the choroid plexus. The left lung adherent at its anterior part; it did not collapse on the chest being opened; its posterior part vascular; its bronchia loaded with frothy serum; no induration except at one spot of its anterior part, where was some coagulable lymph on its pleura; the mucous membrane of the bronchi not vascular; no blood in the lung; black matter, but no serum in the cellular texture and bronchial glands; the right lung firmly adherent to the chest and pericardium; all its lobes consolidated, like *pudding-stone,* rather shrunken, *not an air-cell or blood-vessel visible;* no distinct tubercle. The disease had been allowed to work its disorganising effects without control. No serum in the chest; some in the pericardium.—The heart pale and flabby.

The serous effusion into the air-cells of the left lung was the immediate cause of death; but *simple* inflammation will not explain the remarkable induration here seen; *hepatisation* does not express this condition of the lung; it is truly a scirrhous state, a peculiar phthisical induration.—Ulceration is not an essential component part of the phthisical disorganisation.—The malignant tumor in the head shows that there was a specific cachectic action and disposition in the constitution; a peculiar mode of vascular action, conjoined with a specific state of the blood." 156.

Before we conclude our notice of Dr. Blackmore's cases, it is worth mentioning that they all agree in one particular; a circumstance, too, which no doubt will be very consoling to consumptive patients, and to young medical men, who may be tempted to look into the book for instructions to direct them in practice; we mean their universal fatality. How does this harmonize with the good things promised in the title—" A practical Treatise on the Forms, Causes, *Sanability*, and *Treatment* of Pulmonary Consumption"? The other general remark which we have to make is that, throughout the whole treatise, the name of the stethoscope, the wonder-working safety-tube of the immortal Laennec, is scarcely even mentioned by the Doctor! Attend to the meagre and niggardly notices in the cases which, we suppose, were under the author's care;—" *A full inspiration easily made, and respiratory sound heard well by the stethoscope; no pain.*" " *By the stethoscope a strong* jarring *action of the heart is heard throughout the chest, respiration natural.*" Such is the tribute paid to the greatest medical discovery of modern times!!

So great is the want of discrimination displayed in one case that we are tempted to quote it, as a summing up of our censures on the present work, which the Doctor will, no doubt, think much too severe.

" CASE XXXI.—Pearse, m. a. 42, (Feb. 9th, 1831,) a sailor, of intemperate and idle habits, has been ill for six months; at first he had cough and spitting for a month, which subsided without medical aid. On Christmas eve last he spat a cup-full of blood; blood was then abstracted by a Surgeon-Apothecary, who did not see him again. Some medicines were afterwards taken from a Quack; cough and sputa, not copious, remained. He has now a sallow anxious countenance, with cough, dyspnœa, and occasional hæmoptysis. It was his habit nine months ago to eat only a potatoe and some bread in the day—spirits being his chief aliment. March 21. Hæmorrhoidal tumors painful; much dyspnœa, some cough and viscid sputa, some pain at the chest a few days ago ; recumbent posture untenable ; pulse nearly gone, much anxiety and sleeplessness, costiveness, no dropsy, no fever. At night he eat a beef-steak, and died from suffocation in five hours. The cura to March 23rd, consisted of Miss. sang. 10 oz. empl. canth. duo. emp. picis cum antim. tart. pil. hydrarg. scilla, ipecac. digitalis, colchic. hyosc. ether. nitric. antim. tart. cathart. copaiba ad anum. quinine.

*Inspection*, 12 hours p.m.—The body very warm; no sugillation, but black dissolved blood issued from the incised integuments and muscles. The peritoneum thickened and opake, containing 10 oz. of serum. The left lobe of the liver extended for two inches below the ribs ; the whole organ was enlarged and hardish ; on a section of the left half a dark nutmeg structure was seen, as if the sanguine-vascular portion was turgid ; the back part of the right lobe was more natural. Dark bile in the gall-bladder. The stomach small, its muscular coat enlarged, the veins betwixt its peritoneal and mucous coats as large as a crow-quill ; the mucous coat like scarlet cloth, thickened, rugose, granular, containing viscid-yellow-brown acrid mucus. The intestines were very small, their coats thick ; the valvulæ conniventes of the mucous coat intensely red, covered with acrid bilious mucus ; the peritoneal membrane of a diffuse florid vascularity. The omenta vascular ; the mesentery large and vascular. The pancreas large and hardish. The spleen very small, hard and dark. All the digestive organs shewed severe marks of vascular excitement, and constant contraction ;—the sad effects of habitual drunkenness ! There was no chyme seen ; the last meal must have been digested. In the sacs of the pleuræ little serum ; some in the pericardium. The right lung unadherent, turgid with serum, mucus and air, carneous and vascular at the thick part of its upper lobe ; the interior of the bronchi vascular. The left lung similar to the right, but not carneous ; its air-cells and cellular tissue dropsical. The *heart* as large as a bullock's ; on the under surface of the *left* ventricle an opake jelly-like spot, as if it had been the seat of inflammation ; its muscular substance and cavities vastly enlarged ; the right less so. All the cavities and the large vessels, engorged with black grumous blood. —The aorta and pulmonary arteries very large, inelastic like leather—no osseous granules; the valves sound.

The disease in the aorta may have been the primary affection in the chest ; the enlargement of the heart was from idiopathic inflammation, by the constant stimulus of spirits, and also from the difficult transmission of the blood through the aorta and its abdominal branches. The pulse did not indicate the state of the heart.

The morbid condition of the lung was secondary, and the effect of obstructed circulation in the heart.

Alcohol seems to effect a dissolution of the blood ; its albumen is coagulated, and the serum extravasated on the circulation being impeded ; the glandular organs become indurated, the hollow muscular system contracted and morbidly vascular.

The dropsy of the lungs, which was the chief morbid appearance in them, was not indicated by external dropsy." 93.

We put two simple queries to the Doctor :—1. Is the above a case of consumption at all ? 2. Did you even suspect disease of the heart, which, we are told, was found on dissection " as large as a bullock's." ?

However Dr. Blackmore may resent the strictures which we have deemed it incumbent on us to make on the publication under review, we are convinced that, ten years hence, he will consider them as salutary, although very unpalatable. Nothing is so injurious to a young author as undeserved praise—nothing so profitable as censure, *if* deserved. Dr. B. will say *if*—and console himself with the reflection that our criticisms are unfair, uncandid, and ill-natured. The public will judge.

